# Validity of the “Streitberger” Needle in a Chinese Population with Acupuncture: A Randomized, Single-Blinded, and Crossover Pilot Study

**DOI:** 10.1155/2013/251603

**Published:** 2013-08-01

**Authors:** Chang-cai Xie, Xiu-yun Wen, Li Jiang, Min-jun Xie, Wen Bin Fu

**Affiliations:** ^1^Department of Acupuncture and Moxibustion, Guangdong Provincial Hospital of TCM, Guangzhou 510120, China; ^2^The Second Clinical Medical College, Guangzhou University of Chinese Medicine, Guangzhou 510405, China; ^3^Department of Urology, Guangdong Provincial Hospital of TCM, Guangzhou 510120, China

## Abstract

We studied the validity of a “Streitberger” needle as a valid approach in a Chinese population with experience of acupuncture. Volunteers were recruited from students of the School of Acupuncture and Moxibustion, Guangzhou University of Chinese Medicine. Sixty students receiving education in acupuncture theory and experience in practical acupuncture were tested in study determining whether needling with the placebo needle felt any different from conventional acupuncture. Outcomes included measures of penetration sensation, VAS ratings, and *Deqi* sensation questionnaire. As a result, needle penetration, VAS ratings for either needle and *Deqi* sensation were not significantly different between two kinds of needles. Our findings show that the use of “Streitberger” needle is credible in a Chinese population with acupuncture experience.

## 1. Background

The placebo needle, also referred to as “Streitberger” needle, was developed by Streitberger and Kleinhenz [1]. The nonpenetrating placebo needle has been regarded as a reliable control treatment in the field of acupuncture clinical research [2–4]. Many studies have demonstrated that the sham needle is a convincing therapy for the majority of participants in acupuncture treatment [1, 5].

However, in China, this kind of sham needle has not yet been found in any clinical reports related to the current study. Before this needle is adopted into therapeutic use among Chinese people, validation of the approach clearly needs to be undertaken. For example, a key question that we would like to address is do different people achieve similar results with two different needles? A key objective of this study was to address this question.

## 2. Materials and Methods

### 2.1. The Placebo Needle

**Figure 1:**
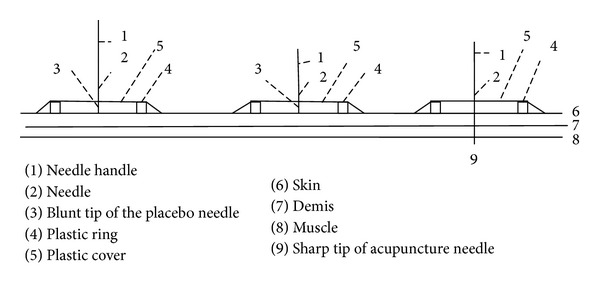
Placebo needle.

For this study, Streitberger designed a special placebo needle. The School of Chinese Medicine of Australia's Royal Melbourne Institute of Technology University provided the needles. Our observations indicated that as the needle was pushed against the skin, it caused a pricking sensation. However, as increased pressure is applied, the shaft of the needle disappears into the handle, which gives the impression that the needle is actually entering the skin. The needle is held in position by a small adhesive plastic ring (Figure 1), which can also be used with the real needles to aid in consistency and credibility. In Streitberger's research, it was reported that none of the volunteers suspected that the needle might not have penetrated the skin [1]. As to the real needle, it had the appearance of the placebo needle, so that participants and manipulators could not distinguish them from their appearance. The only part that differed was that the tip of the real needle was acutely sharper. 

Figure 1 is quoted directly from the article “Introducing a placebo needle into acupuncture research” written by Streitberger and Kleinhenz [1].

### 2.2. Participants

We recruited 40 female and 20 male volunteers aged 22–25, who were students from the School of Acupuncture and Moxibustion of Guangzhou University of Chinese Medicine. All subjects had knowledge of acupuncture and meridians and previous experience in acupuncture. Subjects had no sign of disease and were otherwise healthy. All subjects were willing to take part in this trial. Subjects who had acute or chronic pain, those taking analgesics or psychotropic drugs, and those with skin disease at the site of the acupoint; those who were pregnant were excluded. Ethical approval was obtained from the Ethics Commission of the Guangdong Provincial Hospital of Traditional Chinese Medicine (no. 2008GL-27). Since the majority of people in China have experience in acupuncture, the true validity of a placebo intervention would be more challenging because of their sensation of needles. For this reason, we choose volunteers with previous experience in acupuncture to participate in this research.

### 2.3. Protocols

**Figure 2:**
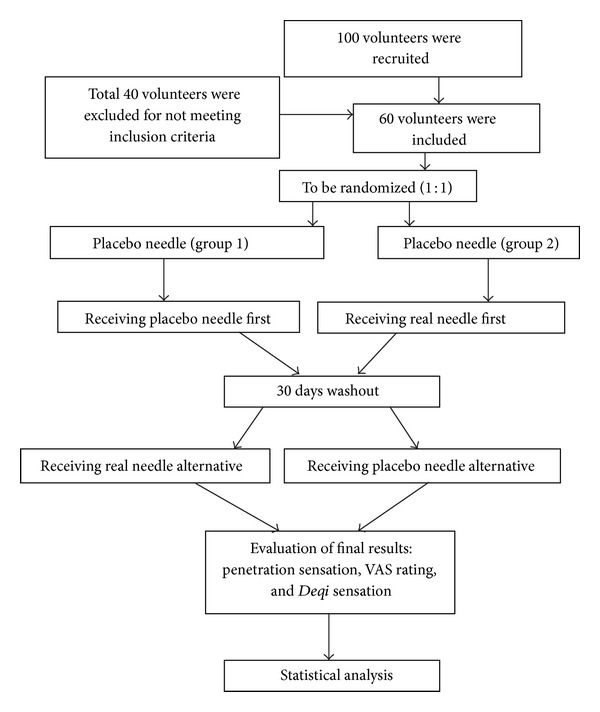
Study protocol.

The trials were conducted in an air-conditioned (22 ± 3°C) room. Volunteers were told that we were testing a new needle to determine if it was more or less painful than a traditional needle. After randomization by using SPSS18.0 statistical software, subjects were treated by acupuncture in a crossover design with acupuncture needling and placebo at Shenshu (BL23). Volunteers took up a prone position during the treatment procedures. Participants could not see the process of the operation since the selected point was located on the back, which might also aid in the blinded design of this pilot study. A total of 30 volunteers were first punctured with placebo acupuncture, 30 others with the placebo needle and vice versa after a 30-day washout or recovery period. The point of acupuncture was disinfected with alcohol and then marked with the plastic ring, which was covered with a plastic sheet. After puncturing the plastic sheet, the needle was depressed. In the case of the real acupuncture, the needle was placed approximately 0.5 cm through the skin; in the case of the placebo acupuncture, this was done until the needle just touched the skin and the shortening of the needle appeared the same as in real acupuncture. The flow chart of the trial is shown in Figure 2. 

Manipulators twirled the needles for 30 sec. After 2 min the needles were removed and the volunteers were asked if they felt the needle penetrating the skin or if the penetration of the needle was painful on a visual analogue scale (VAS) and if they had *Deqi* sensation. 

### 2.4. Statistical Analysis

This study was designed as a crossover trial with each volunteer receiving both the real needles and the placebo needles in sequence. Painful sensations were assessed using VAS ranging from no sensation of pain “not at all or zero point” to “extremely severe ten points” on a sliding scale. As this was a crossover design, a general linear model was used to compare the VAS scores. Responses to the questions relating to needle penetration and *Deqi *sensation were summarized using descriptive statistics and compared using a chi-square test.

## 3. Results

### 3.1. Baseline Data

Groups were well balanced at baseline for gender.  Table 1 shows the division of male and female volunteers as randomized to the two kinds of needles. Distribution of age differed with a *P* value of 0.028 (Table 2). As all the volunteers were students who had accepted education in the practice of acupuncture theory and experience in acupuncture, the statistical differences in the distribution of age would not have affected the outcome of study or of the results.

### 3.2. Outcomes

#### 3.2.1. Needle Penetration

Participants were asked to state whether they felt needle penetration with the real and placebo needles.  Table 3 shows the responses to this question for the 60 participants. 

Of the 60 volunteer subjects, 45 (75.0%) felt that the real needles had penetrated, but 49 (81.7%) felt that the placebo needles had penetrated. 35 (58.3%) felt penetration with both needles, while only 1 subject (1.7%) felt no penetration from either type of needles. Additionally, 14 (23.3%) felt the penetration using placebo needling but did not feel penetration in the case of authentic acupuncture. Further, 10 subjects (16.7%) felt penetration from authentic acupuncture but not from placebo. McNemar's test showed no significant difference. 

#### 3.2.2. VAS Ratings

This was measured in the form of using a VAS. Responses are described in Table 4. The General Linear Model showed an *F* value of 0.217 and a *P* value of 0.643 (*P* > 0.05), which was not statistically significant.

#### 3.2.3. *Deqi*

*Deqi* was sensed by 6 volunteers with acupuncture and placebo needling. Additionally, 8 subjects felt the* Deqi* with placebo needling but did not feel* Deqi* with authentic needling and 18 felt *Deqi* with authentic needling but not with the placebo needle, while 28 subjects did not feel *Deqi* with either approach. Responses are shown in Table 5. McNemar's test showed no statistically significant difference (*P* = 0.076).

## 4. Discussion

Randomised double blind, placebo-controlled trials are considered the gold standard for determining an intervention's specific therapeutic effects. Acupuncture has been tested in randomized controlled trials (RCTs). However, some trials have been criticized for their methodological flaws or limitations. A lack of appropriate placebo or sham controls or sufficient efforts to blind subjects has been key drawback of such studies. Since the introduction of “Streitberger” needles, which were developed by Streitberger and Kleinhenz [1] (Germany), investigators in the field had evaluated its validity in many other countries, such as in the UK and Japan [5, 6]. The Streitberger needle has been used in acupuncture trials as placebo controls [2, 7, 8]. However, such reports are rare in China, and the use of placebo needle and its credibility needs to be tested in more detail.

From the results, penetration between acupuncture and sham needle had no significant difference, and none of the subjects suspected that the skin had not been punctured. *Deqi* was felt by some of the volunteers with placebo needling, but in real acupuncture needling it was felt more often. The pressure of the ring and plastic cover could cause *Deqi* in placebo needling, by psychological influences, or by pain from direct pressure on a pain receptor in the skin. *Deqi* sensation from use of sham needles needs to be verified further. The differences in the VAS ratings between the two needles were also not significant, which means that none of the subjects could recognize use of the sham needle.

However, with regard to the design of this study, the following limitations should be noted. Firstly, this study only had 60 participants, which may not have been a sufficiently large enough sample size, especially for statistical evaluation of the data. Secondly, the healthy college students with previous experience in acupuncture that comprised our study sample population might not have been representative of typical acupuncture patients in the clinical setting. The lack of a strong association between knowledge or experience of acupuncture and correct guessing may have been due to a relatively small number of participants or the homogeneity of the sample in terms of age and educational background. Thirdly, all the volunteers were experienced in acupuncture. Therefore, the results may not be the same among naïve people. Fourth, needle sensation may be different from different acupuncture points on different parts of the body. 

To conclude, nonpenetrating sham needles may serve as a credible sham control in Chinese populations with acupuncture experience. However, the small sample size, varying needle sensations in different acupoints, and the background of participants call for further studies to validate or confirm and/or better understand our findings. Our observations suggest that the “Streitberger” needle is credible to be used in Chinese populations with experience of acupuncture.

## Figures and Tables

**Table 1:** Results of randomization (gender).

Group	Gender		Total
	Male	Female	
Placebo needle	12	18	30
Real needle	8	22	30

Total	20	40	60

**Table 2:** Results of randomization (age).

Group	*N*	Mean	S.D.	S.E.M.
Placebo needle	30	23.13	1.056	0.190
Real needle	30	23.07	0.753	0.140

**Table 3:** Responses of patients to penetration question for the two types of needles.

Placebo needle	Real needle			Total
	Yes	No	No response	
Yes	35	14	—	49 (81.7%)
No	10	1	—	11 (18.3%)
No response	—	—	—	0

Total	45 (75.0%)	15 (25.0%)	0	60

**Table 4:** Tests of between-subjects effects (VAS ratings).

Source	Type III sum of squares	df	Mean square	*F* value	Significance
Intercept					
Hypothesis	449.694	1	449.694	176.303	0.000
Error	150.491	59	2.551^a^		
Stage					
Hypothesis	7.288	1	7.288	2.524	0.118
Error	167.503	58	2.888^b^		
Needle					
Hypothesis	0.628	1	0.628	0.217	0.643
Error	167.503	58	2.888^b^		
Volunteers					
Hypothesis	150.491	59	2.551	0.883	0.682
Error	167.503	58	2.888^b^		

^a^Receiving placebo needle.

^b^Receiving real needle.

**Table 5:** Responses of patients to penetration question for the two types of needles.

Placebo needle	Real needle			Total
	Yes	No	No response	
Yes	6	8	—	14 (23.3%)
No	18	28	—	46 (76.7%)
No response	—	—	—	0

Total	24 (40.0%)	36 (60.0%)	0	60
